# Optimization of Antioxidant Synergy in a Polyherbal Combination by Experimental Design

**DOI:** 10.3390/molecules27134196

**Published:** 2022-06-29

**Authors:** Tsholofelo M. Mapeka, Maxleene Sandasi, Alvaro M. Viljoen, Sandy F. van Vuuren

**Affiliations:** 1 Department of Pharmaceutical Sciences, Tshwane University of Technology, Pretoria 0001, South Africa; mapekat@tut.ac.za (T.M.M.); sandasim@tut.ac.za (M.S.); viljoenam@tut.ac.za (A.M.V.); 2 SAMRC Herbal Drugs Research Unit, Faculty of Science, Tshwane University of Technology, Pretoria 0001, South Africa; 3 Department of Pharmacy and Pharmacology, Faculty of Health Sciences, University of Witwatersrand, Parktown, Johannesburg 2193, South Africa

**Keywords:** culinary herbs, spices, essential oils, combination, antioxidant, design of experiments, MODDE, 2,2-diphenyl-1-picrylhydrazyl, ferric reducing antioxidant power, 2,2-azinobis (3-ethylbenzothiazoline-6-sulphonic acid)

## Abstract

Culinary herbs and spices are known to be good sources of natural antioxidants. Although the antioxidant effects of individual culinary herbs and spices are widely reported, little is known about their effects when used in combination. The current study was therefore undertaken to compare the antioxidant effects of crude extracts and essential oils of some common culinary herbs and spices in various combinations. The antioxidant interactions of 1:1 combinations of the most active individual extracts and essential oils were investigated as well as the optimization of various ratios using the design of experiments (DoE) approach. The 2,2-diphenyl-1-picrylhydrazyl (DPPH), 2,2-azinobis (3-ethylbenzothiazoline-6-sulphonic acid) (ABTS), and ferric reducing antioxidant power (FRAP) assays were used to determine the antioxidant activity, and MODDE 9.1^®^ software (Umetrics AB, Umea, Sweden) was used to determine the DoE. The results revealed synergism for the following combinations: *Mentha piperita* with *Thymus vulgaris* methanol extract (ΣFIC = 0.32 and ΣFIC = 0.15 using the DPPH and FRAP assays, respectively); *Rosmarinus officinalis* with *Syzygium aromaticum* methanol extract (ΣFIC = 0.47 using the FRAP assay); *T. vulgaris* with *Zingiber officinalis* methanol extracts (ΣFIC = 0.19 using the ABTS assay); and *R. officinalis* with *Z. officinalis* dichloromethane extract (ΣFIC = 0.22 using the ABTS assay). The DoE produced a statistically significant (R2 = 0.905 and Q2 = 0.710) model that was able to predict extract combinations with high antioxidant activities, as validated experimentally. The antioxidant activities of the crude extracts from a selection of culinary herbs and spices were improved when in combination, hence creating an innovative opportunity for the future development of supplements for optimum health.

## 1. Introduction

Culinary herbs and spices are an important part of human nutrition, and they have been used for centuries not only as flavoring agents but as food preservatives and for health benefits. Each spice or herb contains many bioactive compounds such as flavonoids, phenolics, sulfur-containing compounds, tannins, alkaloids, vitamins, and essential oils. Some of these compounds are responsible for their reported antioxidant activities [1,2]. Antioxidants are free radical scavengers and thus inhibit lipid peroxidation and other free-radical-mediated processes, protecting the human body from several diseases attributed to the reactions of free radicals [3,4,5,6]. Furthermore, antioxidants are added to food to prevent deterioration through oxidative processes [7,8]. Natural antioxidants, such as those present in culinary herbs and spices, have been investigated as alternatives to synthetic counterparts due to the concerns of potential carcinogenicity and other adverse effects [9,10]. In recent years, various studies have reported on the antioxidant activities of culinary herbs and spices [1,4,8,11,12,13,14,15,16].

Spices and herbs such as *Syzygium aromaticum* (clove), *Mentha piperita* (peppermint), *Cinnamomum zeylanicum* (cinnamon), *Origanum vulgare* (oregano), *Thymus vulgaris* (thyme), *Salvia officinalis* (sage), and *Rosmarinus officinalis* (rosemary), to name a few, were reported to be strong antioxidants due to their high levels of phenolic compounds [5,17,18,19]. Spices and herbs are often added as blended mixtures in culinary preparations. Furthermore, many traditional healing modalities such as the Indian system of medicine (Ayurveda) and Chinese and African traditional medicines rely on combinations of highly complex herbal mixtures to achieve an enhanced effect. Many scientific reports, however, astutely focus on the bioactivity of individual extracts of culinary herbs and spices [11,12,20,21], but their combined effects are not well-documented. The antioxidant interactions of combinations of essential oils from spices and herbs [22,23] and natural antioxidant compounds with synthetic antioxidants [24] have been reported. It could therefore be of great interest to investigate the possible antioxidant interactions occurring when spices are combined. This approach may increase their antioxidant effect at sufficiently low concentrations by taking advantage of their possible synergistic or additive effects. To study the interactions between various spices, herbal extracts, and essential oils requires many experiments to determine which combination ratios provide the optimum antioxidant effect. A design of experiments (DOE) approach was thus utilized to identify and optimize the synergy potential of the extracts, using a limited number of experimental assays, thus saving time and resources. 

Several *in vitro* assays are used to evaluate the antioxidant activities of spices and herb extracts and essential oils, namely, the 2,2-diphenyl-1-picrylhydrazyl (DPPH), oxygen radical absorbance capacity (ORAC), ferric reducing antioxidant power (FRAP), 2,2-azinobis (3-ethylbenzothiazoline-6-sulphonic acid) (ABTS), and microsomal lipid peroxidation (MLP) assays. The present study was therefore designed to evaluate the antioxidant activity of a selection of spices, herb crude extracts, and essential oils by comparatively evaluating the results of three antioxidant assays (DPPH, ABTS, and FRAP) as well as optimizing the synergy potential of the crude extracts and essential oils by investigating the various combinations using the DoE approach.

## 2. Results

### 2.1. Antioxidant Activity of Individual Crude Extracts and Essential Oils

The data on the comparative analysis of the antioxidant activities of crude extracts and essential oils are presented in Table 1. The total antioxidant activity measured by the DPPH assay, presented as half-maximal effective concentration (EC_50_), ranged from 5.48 to 497.10 µg/mL for methanol extracts, 7.66 to 1340.00 µg/mL for the water extracts, 17.85 to 807.80 µg/mL for dichloromethane extracts, and 2.55 to 861.50 µg/mL for the essential oils. The antioxidant properties of a spice or herb differed depending on the solvent used for the extraction. Based on the results in Table 1, *C. zeylanicum* methanol and water extracts demonstrated noteworthy DPPH radical scavenging activities with EC_50_ values of 5.48 and 7.66 µg/mL, respectively. Furthermore, the recorded activity was better than that of the positive control, ascorbic acid (EC_50_ = 10.25 µg/mL). Gupta et al. [25] and Mansour et al. [26] in their studies also reported high DPPH radical scavenging activity for *C. zeylanicum* methanol extracts, though the values were expressed as % DPPH inhibition. Kim et al. [11] reported *C. zeylanicum* water extract to possess a higher DPPH radical scavenging activity compared to other spice extracts. However, the EC_50_ value was higher (0.254 mg/mL) compared to that obtained in this study. The least active extract was *C. sativum* water extract (1340.00 µg/mL).

With regards to the essential oils, noteworthy activity was displayed by *S. aromaticum* oil (EC_50_ = 2.55 µg/mL), followed by *M. piperita* and *C. zeylanicum* oils with EC_50_ values of 6.88 and 7.17 µg/mL, respectively. Other oils displayed moderate to low activities, with *T. vulgaris* essential oil displaying the least antioxidant activity with EC_50_ = 861.50 µg/mL. The antioxidant trend for the ABTS assay was different from the DPPH assay, with the total antioxidant activity ranging from EC_50_ values of 6.06 to 69.19 µg/mL for methanol extracts, 5.79 to 145.90 µg/mL for water extracts, 3.09 to 258.40 µg/mL for dichloromethane extracts, and 5.81 to 1397 µg/mL for the essential oils. The *S. aromaticum* and *Z. officinalis* dichloromethane extracts displayed notable activities with EC_50_ values of 3.09 and 4.15 µg/mL, respectively. 

The activity was higher than that of ascorbic acid (EC_50_ = 4.88 µg/mL). This was followed by *C. zeylanicum* (EC_50_ = 5.95 µg/mL), *O. marjorana* (EC_50_ = 5.79 µg/mL), and *M. officinalis* (EC_50_ = 6.57 µg/mL) water extracts. Methanol extracts of *C. zeylanicum*, *M. piperita*, *Z. officinalis*, *M. officinalis*, *R. officinalis*, and *T. vulgaris* also demonstrated promising ABTS radical scavenging effects with EC_50_ values of 6.06, 6.42, 6.85, 6.94 7.18 and 7.27 µg/mL, respectively. Like the DPPH assay, *S. aromaticum* oil exhibited notable radical scavenging effect with the FRAP assay with EC_50_ = 5.96 µg/mL.

The FRAP assay demonstrated a higher variation compared to the DPPH and ABTS assays, with EC_50_ values ranging from 33.45 to 11,498.00 µg/mL for the methanol extracts and 42.96 to 1143.00 µg/mL for the water extracts. The EC_50_ values for the dichloromethane extracts ranged from 36.26 to 81,083.00 µg/mL, while essential oils ranged from 5.96 to 794.80 µg/mL. The order of active methanol extracts according to the FRAP assay, in comparison to ascorbic acid was *R. officinalis* > *C. zeylanicum* > *M. officinalis* > *T. vulgaris* > *M. piperita* > *S. officinalis* > *S. aromaticum* > *C. citratus* > ascorbic acid. The *C. zeylanicum*, *R. officinalis,* and *S. aromaticum* methanol and dichloromethane extracts showed promising FRAP reducing activities (EC_50_ values = 36.44, 33.45, and 58.47 µg/mL for the methanol extracts and 36.26, 58.92, and 40.95 µg/mL for the dichloromethane extracts, respectively). Furthermore, the *C. zeylanicum* and *R. officinalis* water extracts also demonstrated notable effects (EC_50_ = 42.96 and 45.86 µg/mL, respectively) compared to ascorbic acid (90.59), while *M. officinalis* and *M. piperita* methanol extracts were active at EC_50_ = 39.49 and 53.40 µg/mL, respectively. Other extracts had moderate, low, or no activity with reference to ascorbic acid. In their study, Chan et al. [27] reported the notable FRAP reducing ability of *C. zeylanicum* water extracts. However, the EC_50_ value was higher (0.24 mg/mL) compared to the value obtained in the current study. 

The results on the antioxidant activity of spices or herbal extracts and essential oils demonstrated stronger to very weak correlations among the three assays used in the study (Table 2). A weaker correlation existed between the DPPH and FRAP assays for the water (*r* = 0.42) and methanol (*r* = 0.37) extracts. This is contrary to the findings by Ulewicz-Magulska and Wesolowski [19], who reported a stronger relationship between the two assays (*r* = 0.94). Meanwhile, very weak correlations existed for the dichloromethane extracts (*r* = 0.13) and essential oils (*r* = 0.22). A higher correlation (*r* = 0.71) was shown between the DPPH and ABTS assays for the water extracts. Furthermore, a weak correlation existed between the ABTS and FRAP assays for all the extracts and essential oils. No correlation existed between the DPPH and ABTS assays for methanol extracts.

### 2.2. Interactive Studies

For all the assays, the antioxidant activity of the extracts was compared to that of the positive control. All the extracts and essential oils displaying EC_50_ values closer to the positive control and demonstrating notable antioxidant activity in the three assays were selected for antioxidant interaction studies. Extracts (12) and essential oils (2) were combined (1:1 *v*/*v*) to evaluate the interactive antioxidant activity using the DPPH, ABTS, and FRAP assays., The data results of the comparative analysis of the antioxidant interactions from the different combinations of the extracts and essential oils are presented in Table 3. The results show that 3.80% of the combinations were synergistic in the DPPH assay, and 7.70% were synergistic in the FRAP and ABTS assays, while 50% of the extracts were additive using the DPPH and FRAP assays, and 19.20% were additive with the ABTS assay. Other combinations were indifferent with ABTS (69.20%), and 38.40% were indifferent with the DPPH and FRAP assays. Antagonism was shown for 11.50% of the combinations with DPPH assay and 3.82% for the FRAP assay. None of the combinations were antagonistic -with the ABTS assay. Combining *T. vulgaris* and *Z. officinalis* methanol extracts yielded synergy (ΣFIC = 0.19) in the ABTS assay, while the combination was indifferent using the DPPH and FRAP assays. Synergy was also observed when combining *M. piperita* and *T. vulgaris* methanol extracts using the FRAP (ΣFIC = 0.15) and DPPH assays (ΣFIC = 0.32). Meanwhile these combinations were indifferent in the ABTS assay. Other combinations that were synergistic include *R. officinalis* with *S. aromaticum* methanol extracts at ΣFIC = 0.47 following the FRAP method. However, this combination was additive in the DPPH and ABTS assays. This clearly demonstrates that variability exists between the studied methods, and including different assays, as in this study, provides a better overall assessment of efficacy. 

Similar observations were made when comparing the assays, whereby *M. officinalis* combined with *S. aromaticum* methanol extracts and *M. piperita* with *R. officinalis* methanol extracts displayed additive effects. Meanwhile, the combinations of *R. officinalis* with either *C. zeylanicum* or *Z. officinalis* methanol extracts were indifferent in the three assays. The combinations of *M. piperita* methanol extract with either *C. zeylanicum* or *S. aromaticum* extracts were antagonistic using the DPPH method. A study by Mansour et al. [26] reported synergistic antioxidant effects from a combination of *C. zeylanicum* and *S. aromaticum* methanol extracts with the DPPH assay. Similarly, Purkait et al. [23] reported synergy from combining the two oils using the DPPH assay. However, the combination was indifferent in this study. Saeed et al. [28] also reported synergy from combining *C. zeylanicum* with *S. aromaticum* water extracts using the DPPH assay. Meanwhile, an additive effect was observed in the current study. The difference between their study and the results of this study is that they used the combination index (CI) to evaluate the antioxidant interactions, while in the current study, the ΣFIC was calculated. Furthermore, in their study, synergy was interpreted as any value < 1, while in this study, a more stringent criterion (ΣFIC ≤ 0.5 interpreted as synergy) was used. Overall, the results from the interactive study demonstrated that selected crude extracts from culinary herbs and spices can be combined to achieve synergistic or additive antioxidant effects and thus enhance efficacy. 

### 2.3. Design of Experiments (DOE) Data Analysis and Model Verification

Three extracts (*M. piperita*, *T. vulgaris,* and *Z. officinalis*) were selected for DOE studies based on a synergistic outcome from the combination/interaction studies. This was conducted in order to determine the optimum ratio at which a formulation of the three extracts can be combined to obtain the highest antioxidant effect. Twelve experimental runs with the combinations indicated in Table 4 were obtained from the design, and the EC_50_ values obtained for each combination using the DPPH, ABTS, and FRAP assays were imported into MODDE^®^ 9.0 (Umetrics AB, Umea, Sweden. The data in Table 4 were modeled, and the replication plot, histogram, summary of fit, coefficient plot, residual N- plot, observed versus predicted plot, and the response contour plots were obtained and used to assess the suitability of the PLS model for predictions. 

#### 2.3.1. Summary of Fit Plot

**Figure 1:**
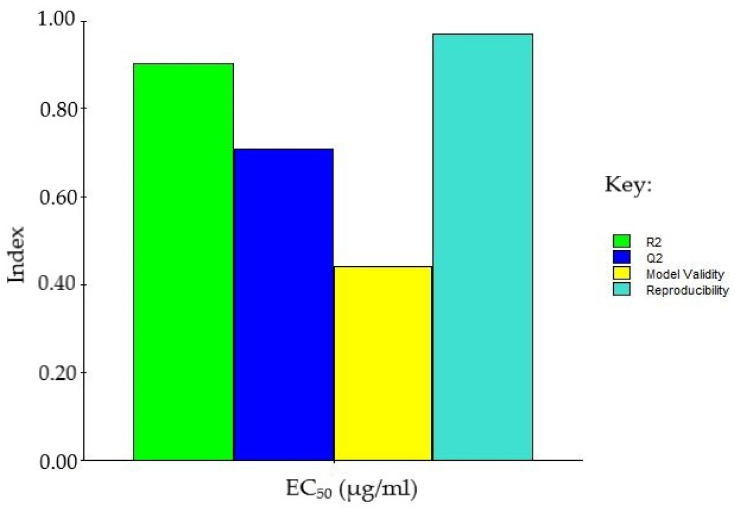
Summary of fit plot for EC_50_ of methanol extract mixtures.

The linear generated model was fitted against the data, and the response is shown in the summary of the fit plot in Figure 1, which provides the information on the strength and robustness of the model. The R2 value (0.91) signified a low variation in the response and a strong fit between the data and the model. Meanwhile, the Q2 value of 0.71 (ideally > 0.5) demonstrated the high predictive power of the model. Furthermore, the model demonstrated a strong validity of 0.44, which is greater than the 0.25 that is required for good models. The model reproducibility of 0.97 is far greater than the requisite value of 0.50, indicating good model design and low error (Figure 1).

#### 2.3.2. Coefficient Plot

**Figure 2:**
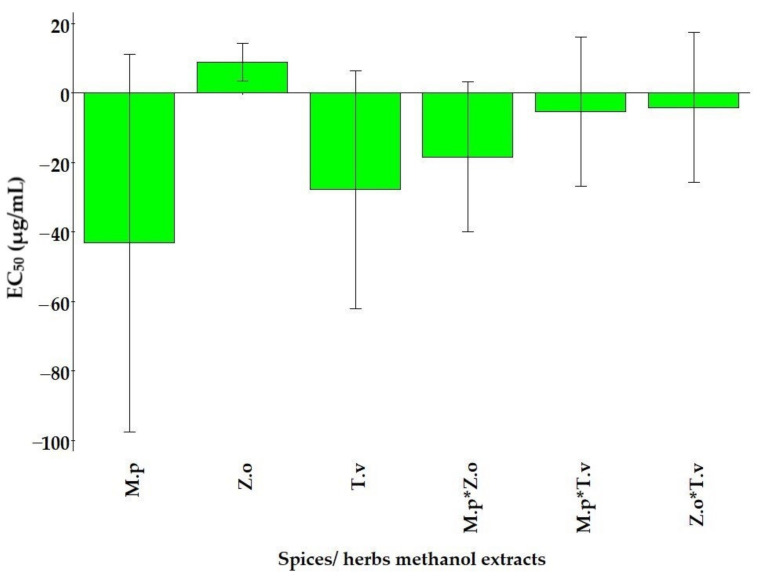
A coefficient plot showing the effects of the three factors *M. piperita* (M.p)*, Z. officinalis* (Z.o), and *T. vulgaris* (T.v) on the antioxidant effect of the mixture (*).

The center-scaled coefficients of each term in the model were used to estimate the significance of the factors to the desired response (EC_50_). It was determined that *M. piperita*, *Z. officinalis,* and *T. vulgaris* methanol extracts and the interactions between *M. piperita* and *Z. officinalis* were important factors to the model outcome, whereas the interactions between *T. vulgaris* with either *M. piperita* or *Z. officinalis* were found to be non-significant factors (Figure 2). Typically, large regression coefficients represent factors with a large contribution to the model response, such as *M. piperita* extract, while regressions with a positive number denote a positive contribution towards the response, such as *Z. officinalis* extract, and a negative number denotes a negative response. 

#### 2.3.3. Response Contour Plot

**Figure 3:**
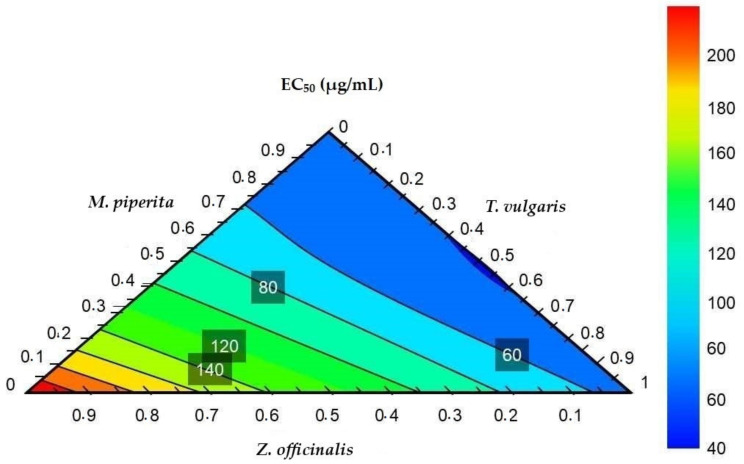
Response contour plot.

The response contour plot allows for the identification of ratios of the combinations that demonstrate the best and worst overall antioxidant effects (Figure 3). The generated model predicted various ratios at which spice/herb extracts can be combined to produce an optimal antioxidant effect. Extract combinations in the red region were predicted to produce high EC_50_ values and, thus, low antioxidant activity. These combinations generally comprise higher proportions of *Z. officinalis* and *T. vulgaris*, and low *M. piperita*. On the opposite end of the color spectrum, combinations in the blue region were predicted to produce lower EC_50_ values and hence higher antioxidant activity. In the blue region, predicted EC_50_ values are given with ratios of combinations with high *M. piperita*, low *T. vulgaris,* and a wider range of *Z. officinalis* content. The optimizer function was then used to determine the best ratio at which the extracts should be combined to obtain the optimum synergistic antioxidant effects. Five combinations in the blue region of the response contour plot were selected, as displayed in Table 4, to validate the model predictions. The DPPH, ABTS, and FRAP methods were used for the model design. However, the FRAP method was chosen, as it produced the best model predictions compared to the DPPH and ABTS methods.

In this study, a statistically significant model was produced, which provided the best combination ratios of the extracts, using limited time and resources. The optimizer showed the optimal antioxidant activity was predicted with a mixture of *M. piperita* (55.00%), *T. vulgaris* (44.00%), and *Z. officinalis* (1.00 %) at EC_50_ = 39.59 µg/mL using the FRAP method. The results from the validation experiments confirmed the reliability and fitness of the model because the correlation coefficient (*r* = 0.7594), calculated using Microsoft Excel^®^ 2019, [29] between the experimental and predicted EC_50_ values for all the combinations (Table 5).

## 3. Materials and Methods

Seventeen commonly used culinary herbs and spices with documented antioxidant activities (Table 6) were selected for this study. Spices were purchased from Warren Chemical Specialties (Pty) Ltd. (Johannesburg, South Africa). The identification was based on the supplier product labelling, as the products were obtained commercially in powder form for all the spices. The materials were kept in a cool and dry place before extraction. Extracts of different polarities (water, methanol, and dichloromethane) were prepared for each of the herbs and spice samples by macerating the preparations in the solvents at a 1:10 solvent ratio, followed by shaking in the dark for 24 h at room temperature using a mechanical shaker. The mixtures were filtered through Whatman No.1 filter paper. The filtrates were then evaporated to dryness under vacuum at 40 °C in a vacuum evaporator (H50- 500, Magna Analytical, Labtech, South Africa). The 17 essential oils were purchased from Prana Monde (Belgium). All commercial essential oils were accompanied by a certificate of analysis. The chromatographic profiling was performed in-house and the marker compounds were identified for each species. The stock solutions of the extracts (1 mg/mL) were prepared by dissolving a known amount of the sample in either methanol, water, or dichloromethane. The solutions were stored at 4 °C until use. The working concentrations (500, 250, 125, 62.5, 31.25, 15.62, 7.81, and 3.91 µg/mL) were prepared from the stock solutions by diluting with the correct volume of methanol. The positive control, ascorbic acid, was prepared in methanol.

The individual extracts and essential oils (Table 6) were screened for antioxidant activity using the DPPH, ABTS, and FRAP assays. The three assays were employed to provide broader information on the antioxidant activity of the tested extracts and essential oils. Measurements were obtained in triplicate for each sample in each assay. The EC_50_ values were calculated for the control and samples, representing the antioxidant capacity in the sample necessary for 50% of the maximal antioxidant effect. In all three assays, the EC_50_ values were determined using GraphPad Prism software, version 5.0 (GraphPad software Inc., San Diego, CA, USA).

### 3.1. The 2,2-Diphenyl-1-picrylhydrazyl (DPPH) Radical Scavenging Assay

The DPPH radical scavenging effects of the extracts and essential oils were estimated according to the method of Brand-Williams et al. [50], with some modifications. Briefly, a DPPH (Sigma-Aldrich, Germany) solution (0.1 Mm *w*/*v*) was prepared in methanol, and 100 µL of the DPPH solution was mixed with 100 µL of the sample in each of the wells of a 96-well microtiter plate. For the DPPH and methanol controls, a volume of 200 µL of DPPH (96 µM in HPLC-grade methanol (Merck, South Africa)) and 200 µL of HPLC-grade methanol were added to the corresponding wells, respectively. Ascorbic acid (22.5 µg/mL) was used as a positive control. The plates were then shaken at 960 rpm for 2 min and incubated in the dark at 27 °C for 30 min. After incubation, the absorbance was read at a single wavelength of 517 nm using a microplate reader (SpectraMax M2 Multimode Microplate Reader, Molecular Devices Inc., USA) linked to a computer with SoftMax^®^ Pro version 6.5.1 for data acquisition and analysis. The inhibition of the DPPH radical by the active samples was determined by calculating the DPPH free radical scavenging activity percentage according to Equation (1).

% Decolorization = 100 × [(A_control_) − (A_test_ + A_methanol_)]/A_control_ (1)

where A = absorbance at 517 nm;
A_control_ = average absorbance of DPPH − average absorbance of methanol;
A_methanol_ = average absorbance obtained in the wells containing methanol;
A_test_ = absorbance obtained in the wells containing DPPH and the test sample.

### 3.2. The 2,2′-Azino-Bis (3-Ethylbenzothiazoline-6-Sulphonic Acid) (ABTS) Cation Radical Scavenging Assay

The spices and herbs were tested for their ABTS radical scavenging activity according to the method of Re et al. [51], with some modifications. Two stock solutions of 7 mM ABTS (Sigma-Aldrich, Burlington, MA, USA) in double-distilled water and 2.45 mM potassium persulphate (Merck, Lethabong, South Africa) were mixed. The mixture was incubated in the dark for 12–16 h at room temperature and used as a working solution. The solution was adjusted with cold ethanol to obtain an absorbance of 0.700 (±0.02) at 732 nm using a microplate reader (Spectra Max M2, Molecular Devices Inc., Silicon Valley, CA, USA). A 100 µL volume of ABTS+ solution was added to the microtiter plate wells containing 100 µL of crude extracts or essential oils. After 30 min of incubation, the percentage of decolorization of ABTS+ at 734 nm was calculated for each concentration relative to the blank, according to Equation (2). Ascorbic acid (22.5 µg/mL) was used as a positive control.

% Decolorization = 100 × [(A_control_) − (A_test_ + A_methanol_)]/A_control_ (2)

where A = absorbance at 734 nm;
A_control_ = average absorbance of ABST^+^ − average absorbance of methanol;
A_methanol_ = average absorbance obtained in the wells containing methanol;
A_test_ = average absorbance obtained in the wells containing ABST^+^ and the test sample.

### 3.3. Ferric Iron-Reducing Antioxidant (FRAP) Power Assay

The ferric iron-reducing antioxidant (FRAP) assay is based on the ability of the antioxidant to reduce Fe^3+^ to Fe^2+^ in the presence of 2,4,6-Tris(2-pyridyl)-1,3,5-triazine (TPTZ), forming an intense blue Fe^2+^-TPTZ [52]. The FRAP reagent was freshly prepared before each experiment by mixing 300 mM acetate buffer (pH 3.6), 20 mM ferric chloride in distilled water, and 10 mM TPTZ (Sigma-Aldrich, Switzerland, in 40 mM HCl) in a ratio of 10:1:1. The FRAP solution (100 µL) was mixed with 100 µL of the test samples and standard solution in each of the wells of a 96-well microtiter plate. Following the same procedure, a blank test containing methanol instead of the extract was used as a negative control, while ascorbic acid at 22.5 µg/mL served as the positive control. The reaction mixtures were then incubated in the dark for 30 min, and the reduction of the Fe^3+^-TPTZ complex to a colored Fe^2+^-TPTZ complex by the extracts and essential oils was monitored by measuring the absorbance after 4 min of incubation at 593 nm using a microplate reader (SpectraMax M2, Molecular Devices Inc., USA). The FRAP value of each sample was calculated using Equation (3) and was expressed as µg/mL ascorbic acid.

FRAP value of sample (µM) = A_sample_ × FRAP value of standard (µM)]/[A_standard_] (3)

where A = absorbance at 593 nm.

### 3.4. Statistical Analysis

A one-way analysis of variance (ANOVA) was performed on each plant extract, and a post hoc Tukey’s multiple comparison test was conducted to calculate the differences between extracts of the same plant. The data from the three antioxidant assays (DPPH, ABTS, and FRAP) were correlated by calculating the Pearson’s correlation coefficient (*r*) using Microsoft Excel^®^ 2019.

### 3.5. Fractional Inhibitory Concentration (FIC)

The sum of the fractional inhibitory concentration index (ΣFIC) was used to measure interactions from different 1:1 combinations of herbs, spices extracts, and essential oils with promising antioxidant effects when tested using the DPPH, FRAP, and ABTS assays. Samples of A and B (50 µL) for the combinations were plated out in each of the wells, which were marked accordingly, followed by the addition of an antioxidant reagent (100 µL) to final concentrations of 3.1 to 500 µg/mL. The ΣFICs for each of the combinations were calculated using Equation (4).

(4) $$\left(\Sigma \mathrm{FIC}\right)=\mathrm{FIC}\;\left(i\right)+\mathrm{FIC}\;\left(ii\right)\;\mathrm{FIC}\;\left(i\right)=\frac{\mathrm{EC}50\;\left(a\right)\;\mathrm{in}\;\mathrm{combination}\;\mathrm{with}\;\left(b\right)}{\mathrm{EC}50\;\left(a\right)\;\mathrm{independently}}\;\mathrm{FIC}\;\left(i\right)=\frac{\mathrm{EC}50\;\left(b\right)\;\mathrm{in}\;\mathrm{combination}\;\mathrm{with}\;\left(a\right)}{\mathrm{EC}50\;\left(b\right)\;\mathrm{independently}}$$

where (a) is the EC_50_ of one spice extract or essential oil in the combination and (b) is the EC_50_ of the other extract or essential oil. The ΣFICs for each combination were interpreted as synergy where the ΣFICs were less than or equal to 0.5 and as additive effects when the ΣFICs were greater than 0.5 but less than or equal to 1.0. For indifference, the ΣFICs were greater than 1.0 but less than or equal to 4.0, and for antagonism, the ΣFICs were greater than 4.0 [53]. For all antioxidant assays, positive and negative controls were included, with a known antioxidant, ascorbic acid (22.5 µg/mL), used as a positive control. Methanol, which was used as a diluent to dissolve the test samples, was used as a negative control.

### 3.6. Experimental Design Using the DOE Model to Determine Effective Antioxidant Combinations

The DoE model was prepared and evaluated using MODDE^®^ version 9.0 (Umetrics AB, Umea, Sweden). The models were fitted with partial least squares (PLS) and were adjusted by removing non-significant terms. To determine which input parameters resulted in the desired outcomes, screening experiments were carried out using a fractional factorial design. Both the independent and dependent variables were fitted to a linear model. This required 12 experimental runs with three center points. All the experiments were completely randomized by the software to reduce bias and experimental errors. After the experimental runs were completed, the results were analyzed using MODDE^®^ version 9.0, and the dataset gave a close-to-normal distribution. Hence, it did not require a logarithmic transformation. A mixture design worksheet for the chosen extracts was produced, and the modeling of responses was generated to confirm the best model fit. A prediction contour plot was generated, which showed the average prediction (point estimate prediction) for every possible combination of the tested extracts. The predictions with desired EC_50_ values were selected for the model validation. These model predictions were then verified by carrying out additional laboratory experiments and comparing the results to the predicted values. 

## 4. Conclusions

The results of this study demonstrated the antioxidant variability between crude extracts and essential oils from culinary herbs and spices using different in vitro methods (DPPH, ABTS, and FRAP assays). The methanol extracts demonstrated better radical scavenging effects compared to the other tested extracts, based on the DPPH and ABTS assays. The *C. zeylanicum* methanol and water extracts were found to be more effective DPPH radical scavengers compared to the standard antioxidant, ascorbic acid. The most superior ABTS radical scavengers were the *S. aromaticum* and *Z. officinalis* dichloromethane extracts, which had greater activity than ascorbic acid. The most effective extracts in reducing ferric iron were the *R. officinalis*, *C. zeylanicum*, *M. officinalis*, *T. vulgaris*, *M. piperita,* and *S. aromaticum* methanol extracts. *Apium graveolens C. zeylanicum, Mentha piperita,* and *S. aromaticum* essential oils were found to be effective antioxidants. The results from the different assays could not be correlated, except for the relationship between the DPPH and FRAP assays for the water extract, which showed a high positive correlation. 

The 1:1 combination displayed synergistic, additive, indifferent, and antagonistic effects. Strong synergism was shown by combining *T. vulgaris* with *Z. officinalis* (methanol extract) using the ABTS assay, *R. officinalis* with *S. aromaticum* (methanol extracts) using the FRAP assay, and from combining *T. vulgaris* and *M. piperita* methanol extracts using the DPPH and FRAP assays. Using the DoE, a model that could easily predict the ratios at which the spice extracts can be combined to achieve the highest antioxidant effect was produced. The use of extracts in various combinations resulted in an optimum antioxidant effect, even at lower concentrations compared to the single extracts due to synergistic interactions. 

## Figures and Tables

**Table 1:** Antioxidant activity of crude extracts and essential oils of some common culinary herbs and spices.

Spice/Herb	Type of Extract	Extraction Yield (%)	DPPH EC_50_ (µg/mL)	ABTS EC_50_ (µg/mL)	FRAP EC_50_ (µg/mL)
*Allium sativum*	Methanol	-	356.90 ± 0.41	47.46 ± 0.11	11,498.00 ± 4.04
	Water	17.10	176.60 ± 0.76 **	142.60 ± 0.15	188.50 ± 0.32 *
	Dichloromethane	0.94	57.98 ± 0.17	19.10 ± 0.16	373.00 ± 0.15 *
	Essential oil		165.80 ± 0.20	159.60 ± 0.02	146.40 ± 0.03
*Anethum graveolens*	Methanol	3.17	96.47 ± 0.04 *	14.29 ± 0.08	454.30 ± 0.17 *
	Water	6.72	144.50 ± 0.22 *	26.82 ± 0.09	192.30 ± 0.99
	Dichloromethane	-	271.90 ± 1.59 ***	129.30 ± 0.10	81,083.00 ± 00 *
	Essential oil		217.60 ± 0.13	194.90 ± 0.54	158.90 ± 0.12
*Apium graveolens*	Methanol	1.41	114.20 ± 0.11	21.47 ± 0.04	784.50 ± 0.17
	Water	18.99	294.80 ± 0.18	87.98 ± 0.05	1143.00 ± 0.40
	Dichloromethane	2.67	235.80 ± 0.99	55.23 ± 0.13	305.90 ± 0.05
	Essential oil		547.30 ± 0.31	42.86 ± 0.12	**69.68** ± 0.11
*Cinnamomum zeylanicum*	Methanol	8.50	**5.48** ± 0.44 ***	**6.06** ± 0.32 *	**36.44** ± 0.06 *
	Water	-	**7.66** ± 0.22 ***	**5.95** ± 0.43 *	**42.96** ± 0.04 **
	Dichloromethane	3.00	287.90 ± 0.55 ***	21.09 ± 0.05 *	**36.26** ± 0.10 **
	Essential oil		7.17 ± 0.17	11.42 ± 0.10	**25.18** ± 0.03
*Coriandrum sativum*	Methanol	1.42	92.41 ± 0.11 **	22.40 ± 0.02	432.70 ± 0.30 *
	Water	5.41	1340.00 ± 3.26 *	111.50 ± 0.09	440.00 ± 0.24 *
	Dichloromethane	7.39	232.80 ± 0.53 *	15.96 ± 0.08	742.70 ± 0.65
	Essential oil		385.00 ± 1.21	1397 ± 3.13	159.70 ± 0.13
*Cymbopogon citratus*	Methanol	0.95	245.10 ± 0.14 *	35.92 ± 0.03	**61.99** ± 0.20 *
	Water	18.23	72.90 ± 0.07 *	29.99 ± 0.04	**79.13** ± 0.19
	Dichloromethane	2.96	807.80 ± 1.68 ***	45.43 ± 0.09	615.30 ± 0.23 *
	Essential oil		601.60 ± 0.53	253.20 ± 0.34	794.80 ± 0.28
*Laurus nobilis*	Methanol	1.61	27.58 ± 0.04	**8.77** ± 0.03 **	120.70 ± 0.06
	Water	12.95	22.53 ± 0.24 ***	17.58 ± 0.05 *	161.40 ± 0.07 ***
	Dichloromethane	3.79	148.40 ± 0.26 ***	47.57 ± 0.07 **	356.30 ± 0.11 ***
	Essential oil		152.40 ± 0.63	49.48 ± 0.06	684.90 ± 1.03
*Melissa officinalis*	Methanol	12.79	17.27 ± 0.05 ***	**6.94** ± 0.40 **	**39.49** ± 0.05 ***
	Water	6.04	117.10 ± 0.08 ***	**6.57** ± 0.25 **	326.20 ± 0.35 ***
	Dichloromethane	4.29	321.00 ± 0.26 ***	68.81 ± 0.13 **	669.80 ± 0.40
	Essential oil		62.38 ± 0.63	73.65 ± 0.07	472.50 ± 0.20
*Mentha piperita*	Methanol	12.00	17.67 ± 0.049 ***	**6.42** ± 0.85	**53.40** ± 0.04 **
	Water	4.99	53.90 ± 0.082 **	19.96 ± 0.03	234.10 ± 0.11 **
	Dichloromethane	3.35	142.60 ± 0.23 ***	125.50 ± 0.12	807.60 ± 0.87 ***
	Essential oil		**6.88** ± 0.13	34.08 ± 0.13	257.40 ± 0.88
*Ocimum bacilicum*	Methanol	1.57	497.10 ± 0.63	19.2 ± 0.03	1090.00 ± 0.55
	Water	9.33	125.70 ± 0.05	53.54 ± 0.08	429.50 ± 1.64
	Dichloromethane	2.99	172.30 ± 0.07	29.48 ± 0.05	323.30 ± 0.15
	Essential oil		309.60 ± 0.37	756.0 ± 3.36	1092.00 ± 1.61
*Origanum marjorana*	Methanol	1.67	35.25 ± 0.11	**8.18** ± 0.03	100.70 ± 0.06
	Water	8.38	32.02 ± 0.21	**5.79** ± 0.40	136.80 ± 0.03
	Dichloromethane	3.40	56.41 ± 0.33	12.30 ± 0.20	158.50 ± 0.04
	Essential oil		524.00 ± 3.42	162.00 ± 0.12	374.80 ± 0.21
*Petroselinum crispum*	Methanol	2.57	365.70 ± 1.09	69.19 ± 0.07 **	**77.35** ± 0.21 *
	Water	5.07	81.83 ± 0.12 *	15.70 ± 0.41 **	136.40 ± 0.12 *
	Dichloromethane	5.99	98.47 ± 0.16 *	258.40 ± 0.41	**81.42** ± 0.47
	Essential oil		15.51 ± 0.15	126.30 ± 0.10	769.60 ± 0.52
*Rosmarinus officinalis*	Methanol	6.20	**11.64** ± 0.05 *	**7.18** ± 0.30	**33.45** ± 0.13 *
	Water	5.62	36.15 ± 0.12 *	10.56 ± 0.21	**45.86** ± 0.11 *
	Dichloromethane	2.69	17.85 ± 0.03	16.78 ± 0.15	**58.92** ± 0.17
	Essential oil		444.30 ± 0.58	195.90 ± 0.24	484.10 ± 0.58
*Syzygium aromaticum*	Methanol	11.2	**11.07** ± 0.05	10.21 ± 0.04	**58.47** ± 0.15
	Water	10.34	18.91 ± 0.07	**8.43** ± 0.17	102.10 ± 0.26
	Dichloromethane	5.24	28.96 ± 0.05	**3.09** ± 1.89	**40.95** ± 0.19
	Essential oil		**2.55** ± 0.40	**5.81** ± 1.35	**5.96** ± 0.71
*Salvia officinalis*	Methanol	1.49	40.16 ± 0.03	16.36 ± 0.03	**58.31** ± 0.03
	Water	8.90	39.03 ± 0.03	17.18 ± 0.06	223.50 ± 0.01
	Dichloromethane	5.62	35.20 ± 0.05	**8.55** ± 0.12	94.77 ± 0.02
	Essential oil		59.07 ± 0.07	149.10 ± 0.08	171.40 ± 0.04
*Thymus vulgaris*	Methanol	9.40	32.73 ± 0.04	**7.27** ± 0.17	**45.10** ± 0.05 *
	Water	12.08	135.80 ± 0.57	27.48 ± 0.05	249.80 ± 0.09 *
	Dichloromethane	4.82	74.30 ± 0.05	16.01 ± 0.04	233.20 ± 0.35
	Essential oil		861.50 ± 5.05	147.90 ± 0.09	537.80 ± 0.85
*Zingiber officinalis*	Methanol	-	22.18 ± 0.05 ***	**6.85** ± 0.25	220.00 ± 0.02 *
	Water	9.03	893.60 ± 0.11 ***	145.90 ± 0.33	569.50 ± 0.34 *
	Dichloromethane	5.63	30.41 ± 0.05 ***	**4.15** ± 0.57	133.60 ± 0.11 *
	Essential oil		129.40 ± 0.26	275 ± 0.82	363.90 ± 0.24
	Positive control		10.25	4.88	90.59
	Negative control		>500	>500	>500

Values represent means ± standard deviations for experiments performed in triplicate. Values in bold indicate noteworthy antioxidant activity in comparison with the positive control, ascorbic acid. The lower the EC_50_ value, the higher the antioxidant activity. Extracts from same spice/herb with significant differences are shown with * (*p* ≤ 0.05), ** (*p* ≤ 0.01), and *** (*p* ≤ 0.001).

**Table 2:** Pearson’s correlation between antioxidant activity of solvent extracts by different antioxidant assays.

Type of Extract	DPPH/FRAP (*r*)	DPPH/ABST (*r*)	FRAP/ABST (*r*)
Methanol	0.37	0.005	0.20
Water	0.42	0.71	0.48
Dichloromethane	0.13	0.10	0.31
Essential oil	0.23	0.22	0.16

**Table 3:** Antioxidant effects from the combinations (1:1 *v*/*v*) of active extracts and essential oils with the DPPH, ABTS, and FRAP assays.

Extracts/Essential Oils	DPPH Assay		ABTS Assay		FRAP Assay	
	ΣFIC	Interpretation	ΣFIC	Interpretation	ΣFIC	Interpretation
*C. zeylanicum* + *M. officinalis* methanol	0.99	ADD	1.55	I	0.87	ADD
*C. zeylanicum* + *M. piperita* methanol	30.13	A	1.70	I	0.87	ADD
*C. zeylanicum* + *R. officinalis* methanol	2.46	I	1.72	I	2.42	I
*C. zeylanicum* + *S. aromaticum* methanol	2.15	I	1.09	I	0.93	ADD
*C. zeylanicum* + *T. vulgaris* methanol	0.95	ADD	2.29	I	1.14	I
*C. zeylanicum* + *Z. officinalis* methanol	1.94	I	1.28	I	0.62	ADD
*M. officinalis* + *M. piperita* methanol	0.87	ADD	1.19	I	2.36	I
*M. officinalis* + *R. officinalis* methanol	1.36	I	1.12	I	3.46	I
*M. officinalis* + *S. aromaticum* methanol	0.92	ADD	0.83	ADD	0.55	ADD
*M. officinalis* + *T. vulgaris* methanol	0.72	ADD	1.12	I	0.79	ADD
*M. officinalis* + *Z. officinalis* methanol	1.39	I	1.65	I	0.83	ADD
*M. piperita* + *R. officinalis* methanol	0.51	ADD	0.55	ADD	0.90	ADD
*M. piperita* + *S. aromaticum* methanol	16.93	A	1.04	I	0.93	ADD
*M. piperita* + *T. vulgaris* methanol	0.32	S	1.03	I	0.15	S
*M. piperita* + *Z. officinalis* methanol	0.54	ADD	1.82	I	0.67	ADD
*R. officinalis* + *S. aromaticum* methanol	0.86	ADD	1.00	ADD	0.47	S
*R. officinalis* + *T. vulgaris* methanol	0.90	ADD	1.16	I	1.19	I
*R. officinalis* + *Z. officinalis* methanol	1.07	I	1.08	I	1.03	I
*S. aromaticum* + *T. vulgaris* methanol	1.04	I	0.96	ADD	1.15	I
*S. aromaticum* + *Z. officinalis* methanol	2.07	I	1.03	I	0.51	ADD
*T. vulgaris* + *Z. officinalis* methanol	1.22	I	0.19	S	1.28	I
*C. zeylanicum* + *S. aromaticum* water	0.78	ADD	1.48	I	1.49	I
*R. officinalis* + *S. aromaticum* dichloromethane	0.79	ADD	0.84	ADD	1.17	I
*R. officinalis* + *Z. officinalis* dichloromethane	0.51	ADD	0.22	S	0.65	ADD
*S. aromaticum* + *Z. officinalis* dichloromethane	1.66	I	2.80	I	0.80	ADD
*C. zeylanicum* + *S. aromaticum* essential oil	18.29	A	4.33	A	21.51	A

ΣFIC: Sum of fractional inhibitory concentration index; ADD: additive; S: synergy; I: Indifferent; A: antagonism.

**Table 4:** The worksheet used to fit the model.

Exp No	Exp Name	Run Order	*M. piperita*	*Z. officinalis*	*T. vulgaris*	EC_50_
1	N1	3	1	0	0	53.40
2	N2	2	0	1	0	220.00
3	N3	8	0	0	1	45.10
4	N4	5	0.67	0.17	0.17	62.48
5	N5	9	0.17	0.67	0.17	146.10
6	N6	6	0.17	0.17	0.67	92.00
7	N7	1	0	0.50	0.50	111.90
8	N8	11	0.50	0	0.50	7.25
9	N9	4	0.50	0.50	0	68.71
10	N10	10	0.33	0.33	0.33	94.50
11	N11	7	0.33	0.33	0.33	96.10
12	N12	12	0.33	0.33	0.33	85.25

**Table 5:** Model validation for experimental runs using the FRAP assay.

Modeled Combination Ratios				
*M. piperita*	*Z. officinalis*	*T. vulgaris*	Predicted	Experimental Validation
0.55	0.01	0.44	39.59	47.61
0.82	0.15	0.02	52.99	57.26
0.71	0.26	0.03	59.34	59.60
0.38	0.08	0.54	46.89	40.87
0.48	0.10	0.42	47.46	47.56

**Table 6:** Common culinary herbs and spices with documented antioxidant activities.

Spice/Herb	Common Name	Type of Extract	Justification for Inclusion in the Study	References
*Allium sativum* L.	Garlic	Ethanol	DPPH radical scavenger at EC_50_ value of 41.00 ± 1.00 (g spice/Kg DPPH)	[30]
*Anethum graveolens* L.	Dill	Ethanol	DPPH radical scavenger at EC_50_ values of 75.00 ±1.00 (g spice/Kg DPPH)	[30,31]
		Methanol, hexane, dichloromethane	Demonstrated DPPH, hydroxyl, nitric oxide and superoxide radical scavenging activity at IC_50_ values ranging from 0.24–1.43 mg/mL	
*Apium* *graveolens* L.	Celery	Methanol, ethanol, hexane	DPPH and ABTS radical scavengers (IC_50_ values ranging from 0.47–1.41 mg/mL), FRAP values ranging from 8.64 to 12.48 mmol/L	[32,33]
		Essential oil	Demonstrated DPPH radical scavenging activity at 56.68% for the seed and 69.30% for the leaves	
*Cinnamomum zeylanicum* Garcin ex Blume	Cinnamon	Aqueous	DPPH radical scavenger at 0.254 mg/ml	[34,35]
		Essential oil	Hydrogen peroxide and nitric oxide scavenging activity at 30.73% and 15.23%, respectively	
*Coriandrum sativum* L.	Coriander	Ethanol	Demonstrated radical scavenging activity at EC_50_ values value of 235.00 ± 5.00 (g spice/Kg DPPH)	[30,36]
		Methanol	Demonstrated DPPH radical scavenging activity at EC_50_ value of 0.18 µg/mL	
*Cymbopogon citratus* (hort. ex DC.) Stapf	Lemongrass	Essential oil	Showed ability to inhibit DPPH radical at 78.89–89.00% and free radical scavenging effect at 44.06 mg Trolox/100mL	[37,38,39]
*Laurus* *nobilis* L.	Bay laurel	Ethanol	DPPH free radical scavenger at EC_50_ value of 3.90 ± 0.20 (g spice/Kg DPPH)	[30,40]
*Melissa* *officinalis* L.	Lemon balm	Methanol	DPPH radical scavenger at IC_50_ value of 20.16 µg/mL	[19,41,42]
		Aqueous	DPPH radical scavenger at 51.60% and 48.10% for the methanol and aqueous extracts, respectively	
		Essential oil	DPPH radical scavenging activity with IC_50_ value of 14.02 µg/mL	
*Mentha x* *piperita* L.	Peppermint	Methanol	DPPH radical scavenger at IC_50_ value of 17.19 µg/mL	[19,43]
			DPPH radical scavenger at 39.90%	
*Ocimum* *basilicum* L.	Basil	Ethanol	DPPH free radical scavenger at EC_50_ values of 43.00 ± 0.20 (g spice/Kg DPPH)	[30,43]
		Methanol	DPPH radical scavenger at IC_50_ value of 41.80 µg/mL	
*Origanum marjorana* L.	Marjoram	Ethanol	Demonstrated DPPH radical scavenging activity at EC_50_ values of 17.84 mg/mL	[30]
*Petroselinum crispum (Mill.)* Nym.ex A.W. Hill	Parsley	Methanol, aqueous, hexane, ethyl acetate, dichloromethane	Demonstrated DPPH radical scavenging activity with IC_50_ values of 3310.00, 4485.00, and 4712.00 µg/mL and ferric reducing power with values ranging from 0.014 – 0.360 mmol g^−1^	[44,45]
*Rosmarinus officinalis* L.	Rosemary	Ethanol, methanol, essential oil	Demonstrated DPPH radical scavenging activity at EC_50_ values of 3.86 ± 0.06 (g spice/Kg DPPH) and IC_50_ values of 15.15 µg/mL	[30,43]
*Syzygium aromaticum *(L.)* Merr. and L.M.Perry*	Clove	Aqueous, essential oil, ethanol, hexane	DPPH radical scavenger with EC_50_ values of ranging from 0.125 – 0.55 µg/mL for the extract and 21.50 µg/mL for the essential oil	[46,47,48]
*Salvia* *officinalis* L.	Sage	Aqueous and ethanol	EC_50_ value of 0.40 ± 0.20 –1.65 ± 0.00 µg/mL by DPPH assay and 42.3 ± 3.10 µg/mL with FRAP assay	[30,43]
		Methanol	Demonstrated DPPH radical scavenging activity at IC_50_ of 15.04 µg/mL	
*Thymus* *vulgaris* L.	Thyme	Ethanol	Demonstrated DPPH radical scavenging activity with EC_50_ value of 6.4 ± 0.3 (g spice/Kg DPPH)	[30,43]
		Methanol	Demonstrated DPPH radical scavenging activity at IC_50_ value of 21.91 µg/mL	
*Zingiber officinalis* Roscoe	Root	Methanol	Demonstrated free radical scavenging activity with IC_50_ values of 14.0 and 67.5 µg/mL with DPPH and ABTS assays, respectively	[30,49]
		Ethanol	Ability to scavenge DPPH free radicals at EC_50_ values of 19.10 ± 0.30 (g spice/Kg DPPH)	
